# Mapping QTL Contributing to Variation in Posterior Lobe Morphology between Strains of *Drosophila melanogaster*

**DOI:** 10.1371/journal.pone.0162573

**Published:** 2016-09-08

**Authors:** Jennifer L. Hackett, Xiaofei Wang, Brittny R. Smith, Stuart J. Macdonald

**Affiliations:** 1 Department of Molecular Biosciences, University of Kansas, 1200 Sunnyside Avenue, Lawrence, Kansas, 66045, United States of America; 2 Center for Computational Biology, University of Kansas, 2030 Becker Drive, Lawrence, Kansas, 66047, United States of America; University of Iceland, ICELAND

## Abstract

Closely-related, and otherwise morphologically similar insect species frequently show striking divergence in the shape and/or size of male genital structures, a phenomenon thought to be driven by sexual selection. Comparative interspecific studies can help elucidate the evolutionary forces acting on genital structures to drive this rapid differentiation. However, genetic dissection of sexual trait divergence between species is frequently hampered by the difficulty generating interspecific recombinants. Intraspecific variation can be leveraged to investigate the genetics of rapidly-evolving sexual traits, and here we carry out a genetic analysis of variation in the posterior lobe within *D*. *melanogaster*. The lobe is a male-specific process emerging from the genital arch of *D*. *melanogaster* and three closely-related species, is essential for copulation, and shows radical divergence in form across species. There is also abundant variation within species in the shape and size of the lobe, and while this variation is considerably more subtle than that seen among species, it nonetheless provides the raw material for QTL mapping. We created an advanced intercross population from a pair of phenotypically-different inbred strains, and after phenotyping and genotyping-by-sequencing the recombinants, mapped several QTL contributing to various measures of lobe morphology. The additional generations of crossing over in our mapping population led to QTL intervals that are smaller than is typical for an F_2_ mapping design. The intervals we map overlap with a pair of lobe QTL we previously identified in an independent mapping cross, potentially suggesting a level of shared genetic control of trait variation. Our QTL additionally implicate a suite of genes that have been shown to contribute to the development of the posterior lobe. These loci are strong candidates to harbor naturally-segregating sites contributing to phenotypic variation within *D*. *melanogaster*, and may also be those contributing to divergence in lobe morphology between species.

## Introduction

**Fig 1:**
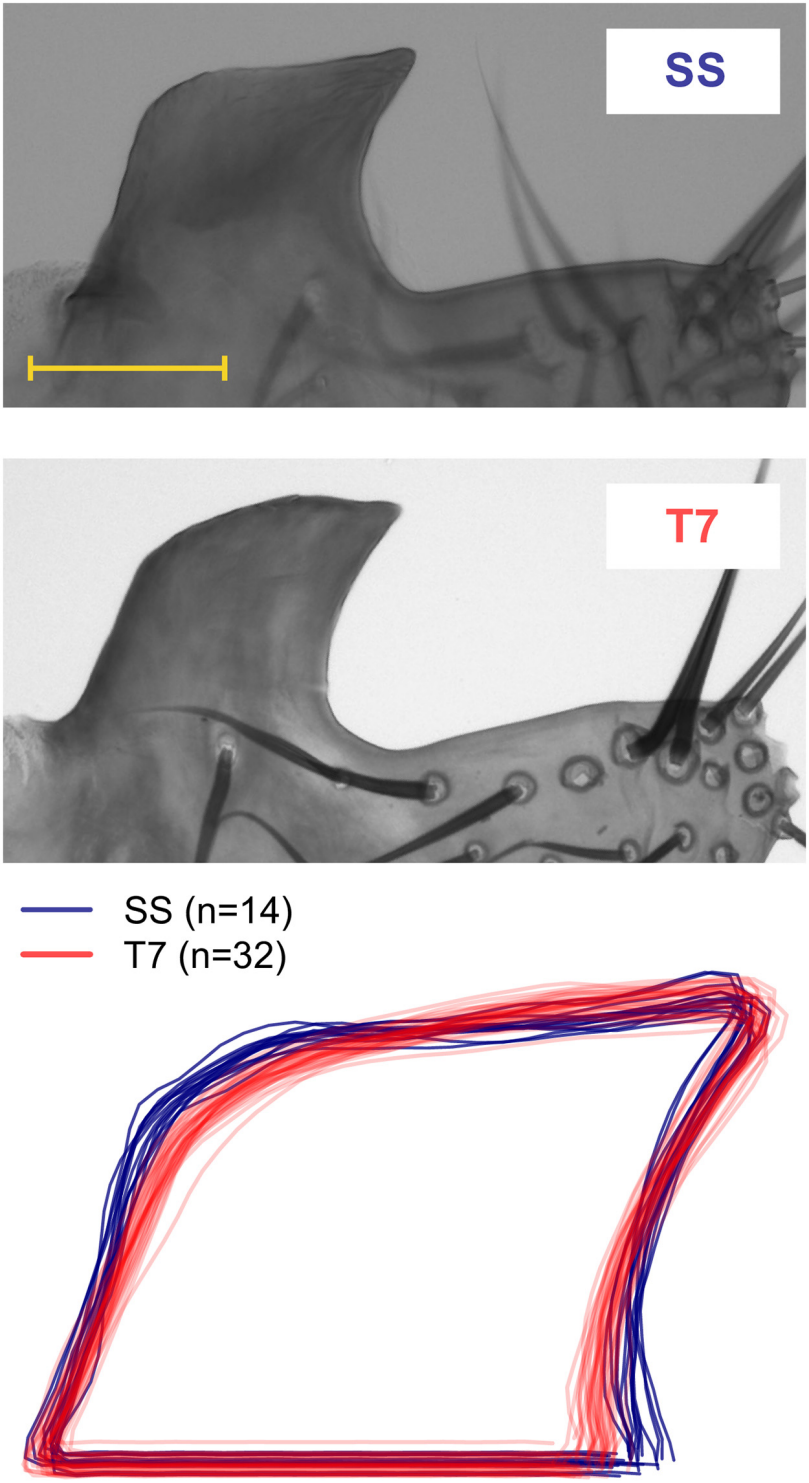
Posterior lobes of parental lines. A representative image of a single posterior lobe is shown for each parental strain. The yellow horizontal scale bar represents 0.05mm. In addition, post-elliptic Fourier analysis reconstructed outlines of all parental lobes are overlaid to highlight the differences in morphology.

The posterior lobe is a male-specific elaboration of the genital arch (Fig 1) present in all four species of the *melanogaster* clade of *Drosophila* (i.e., *D*. *mauritiana*, *D*. *melanogaster*, *D*. *sechellia*, and *D*. *simulans*), and absent in other Drosophilid species, including those of the related *yakuba* clade [1]. The lobe is an evolutionarily novel cuticular structure used by the male to grasp the female ovipositor during mating [1], and both specific laser manipulation of lobe morphology in *D*. *simulans* [2] and genetic ablation of the lobe in *D*. *melanogaster* [3], have indicated the lobe is essential for copulation to occur.

The four species of the *melanogaster* complex are extremely similar morphologically, but differ markedly in the shape and size of the posterior lobe [4, 5]. As such the lobe represents the primary character used to establish species identity in the group. This striking diversity in the lobe mirrors observations from numerous other insect taxa, demonstrating that male genitalia traits are frequently subject to rapid evolution, likely as a result of sexual selection [6, 7]. The precise mechanism(s) by which sexual selection has influenced posterior lobe morphology is unclear. However, behavioral genetics investigations have revealed a range of pre- and post-copulatory effects acting through variation in lobe morphology, suggesting sexual selection may play a role at multiple levels to influence the phenotype [2, 3, 8].

One additional approach to understand the selective forces acting on a trait is to dissect the genetic basis of phenotypic variation to the level of the underlying causative alleles. Describing the genetic architecture of trait variation in this way provides estimates of the effects and frequencies of alleles at quantitative trait loci, QTL [9], from which one can make predictions about the types of process acting to maintain heritability [10, 11]. Cloning the precise causative alleles will ultimately allow exploration of the selective forces acting at causative loci through the application of comparative genome sequencing and tests for patterns of selection [12–14].

Several studies have mapped QTL contributing to the radical divergence among species in posterior lobe morphology using pairwise intercrosses of *D*. *mauritiana*, *D*. *sechellia*, and *D*. *simulans* [15–20]. Exploring genetic differences between *D*. *melanogaster* and its relatives is not possible given the evolutionary divergence between taxa [21], and the resulting inability to generate interspecific recombinants [22]. A challenge for these interspecific mapping studies is the difficulty directly validating putative candidate genes in the species under test, relying instead on functional tests in the *D*. *melanogaster* model system [19]. We and others have instead interrogated standing genetic variation within *D*. *melanogaster* to identify loci contributing to segregating intraspecific variation in lobe shape and size [23, 24]. By using *D*. *melanogaster* we can leverage the wealth of information on gene function in the system to uncover potential candidates [25], and ultimately use sophisticated tools to validate the functional roles of potential lobe loci in the same species in which the QTL were isolated. Under the assumption that the loci contributing to within and between species lobe variation are the same, genes identified within *D*. *melanogaster* as harboring alleles contributing to intraspecific lobe variation, could also be those that have fixed for allelic differences between species (see [26]).

In our previous work we identified three QTL contributing to variation in posterior lobe morphology in *D*. *melanogaster* using a cross between a pair of phenotypically different inbred strains [23]. Here we attempt to replicate these QTL using an independent mapping cross. We take an different pair of highly-inbred, naturally-derived lines showing a difference in lobe morphology, intercross for several generations to expand the genetic map, and identify several QTL that collectively explain a large fraction of the variation in the cross. Two of the QTL mapped by McNeil et al. [23] overlap with QTL mapped in the present study, potentially indicating common genetic underpinnings. In addition, mapped QTL intervals include loci implicated by a genomewide association study, GWAS [24], and genes that have been functionally implicated in the control of genital morphology in *D*. *melanogaster* [19, 27, 28]. These loci are attractive candidates to harbor segregating variation contributing to posterior lobe variation.

## Materials and Methods

### *Drosophila* stocks

We used a pair of highly-inbred *D*. *melanogaster* stocks in this study. Strain "SS" is a version of the *iso1* reference genome sequenced strain, Bloomington *Drosophila* Stock Center number 5027 [29], that was purged of *Wolbachia* via tetracycline treatment. Strain "T7" is a version of *Drosophila* Species Stock Center strain 14021–0231.7 that was originally collected in 1968 from Taiwan, is naturally *Wolbachia*-free, and was further inbred in the Macdonald lab via 18 generations of brother-sister mating [30]. Both SS and T7 are free of *P*-elements, i.e., have the *M* cytotype.

### Mapping population

We generated F_1_ males and females from both reciprocal crosses, i.e., male SS × virgin female T7 (cross A), and male T7 × virgin female SS (cross B). We then generated F_2_ males and females by intercrossing F_1_ animals in all pairwise combinations (i.e., male cross A progeny × virgin female cross A progeny, male cross A progeny × virgin female cross B progeny, male cross B progeny × virgin female cross A progeny, and male cross B progeny × virgin female cross B progeny). All F_2_ animals were mixed, re-distributed into 12 replicate vials to lay eggs, and adults were cleared to maintain roughly similar egg density across vials. Each subsequent generation was treated similarly, using 18–29 replicate vials per generation. No effort was made to collect virgin flies after the F_2_ generation, and generations (egg to adult) were 12–14 days. At the F_13_ generation several hundred recombinant male progeny were frozen at –20°C for phenotyping and genotyping.

All fly maintenance was conducted at 25°C and 50% relative humidity under a 12 hour light: 12 hour dark cycle. Flies were reared in narrow polystyrene vials (25 × 95 mm) containing 10 ml of cornmeal-molasses-yeast medium.

### Lobe dissection and phenotype acquisition

Full details of the dissection and imaging procedures followed are described in McNeil et al. [23], except that in the current study we employed PVA mounting medium (BioQuip catalog number 6371A). Carcasses of dissected males were stored at –20°C in preparation for genotyping.

All high-quality posterior lobe images were manually flipped/rotated to ensure images were of the same handedness, with the "point" of the lobe pointing clockwise. We then employed *ImageJ* [31] to manually outline the lobe, closing the outline with an artificial baseline that extends from the point at which the lateral plate connects to the lobe. Each lobe is thus represented by an outline described by a set of Cartesian coordinates. The coordinates for each lobe were then rotated so that the baseline is horizontal, and translated to center the coordinate series at the centroid of the outline (S1 Fig). This serves to standardize lobes with respect to handedness, orientation, and relative location, allowing comparison of shape across genotypes.

In common with previous investigators [16–18, 23] we employed elliptic Fourier analysis, EFA [32, 33], to describe the shape of each posterior lobe as a series of Fourier coefficients. Since we had already placed each lobe in a standard configuration the additional coefficient normalization routines described in Kuhl & Giardina [33] were not employed. We then used 100 Fourier coefficients for each of the successfully imaged lobes from SS, T7, and the F_13_ recombinant genotypes in a principal components analysis (PCA) to encapsulate shape variation in a small number of quantitative metrics. PCA was carried out using the 'prcomp' function in R (r-project.org), and the principal components (PCs) used as phenotypes for QTL mapping. In addition to PC-based phenotypes we calculated area, height and width (measured as the length of the vertical or horizontal line passing through the centroid and intersecting the outline), and the height:width ratio for each lobe.

### Genotyping-by-sequencing

We extracted DNA from the carcasses of 192 recombinant F_13_ males, and from the SS and T7 parental strains, using the Puregene cell and tissue kit (Qiagen) following the manufacturer's protocol, and subsequently used two different strategies to collect genomewide genotyping data.

Half of the phenotyped F_13_ males, along with both parental genotypes, were genotyped via low pass whole genome sequencing (WGS). Briefly, we mechanically sheared genomic DNA isolated from each sample (Covaris S220), quantified the amount of sheared DNA using the Qubit high sensitivity double-stranded DNA kit (ThermoFisher), and generated indexed sequencing libraries from ~60ng of sheared DNA (New England Biolabs, NEBNext E6040L). Libraries were combined into four 24-plex pools, and each pool was run over a single lane of an Illumina HiSeq2500 sequencer (KU Genome Sequencing Core facility) generating paired-end 100bp reads. Data is available on Dryad (doi:10.5061/dryad.gc182).

The other half of the recombinant males, along with additional samples from both parental genotypes, were genotyped by the MSG (multiplexed shotgun genotyping) method of Andolfatto et al. [34]. Briefly, 10ng of genomic DNA from each fly was digested using a restriction enzyme, and barcoded adaptors were ligated onto each fragmented sample. Multiple samples were then pooled, and each pool was purified, size-selected, and PCR amplified to generate an indexed, sequenceable library. Individual samples are then jointly distinguished by an "in line barcode" (the first 6 nucleotides of the Read1, or "forward" sequencing read) and a standard index read sequence. For reasons unrelated to the current project, we subjected the same 96 samples to MSG using three experimental protocols, using different restriction enzymes (*MseI* and *NdeI*) and reaction conditions. Reads resulting from all three regimes were pooled for each individual prior to genotype calling. MSG libraries were sequenced over a fraction of a HiSeq2500 lane resulting in single-end 100bp reads. Data is available on Dryad (doi:10.5061/dryad.gc182).

There was no association between sample phenotype and the method employed to collect genotypes. Simple *t*-tests contrasting the trait values of those recombinants subjected to WGS- or MSG-based genotyping were not significant in most cases (*p* > 0.1 for lobe area, height, width, height-to-width ratio, PC2, and PC3), and only nominally significant in one case that does not survive correction for multiple testing (*p* = 0.045 for PC1).

### Genotype calling

Sequencing reads were first de-multiplexed via indices (WGS and MSG datasets) and barcode sequences (MSG dataset only), and raw reads were preprocessed using Scythe (version 0.991, github.com/vsbuffalo/scythe) to remove adaptors, and Sickle (version 1.200, github.com/najoshi/sickle) to trim low-quality bases/reads. Filtering resulted in a median of ~572,000 single-end reads for each sample subjected to MSG, and ~6.2 million paired reads for most WGS samples (eight WGS samples experienced problems during sequencing resulting in the collection of only single-end data).

Filtered reads were assembled to a repeatmasked version of the *D*. *melanogaster* genome (release 6.03) consisting solely of the major chromosome arms (X, 2L, 2R, 3L, 3R, 4) using BWA with default settings [35]. Next, data from all genotyped samples—all recombinants, and both parental strains SS and T7—was passed through GATK [36] to identify putative SNP differences between the parents that segregate in the mapping panel.

GATK yielded a set of 692,603 putative SNPs in a VCF file, and we used a custom Python script to retain only those SNPs that passed a series of quality filters (adapted from [37]). Specifically, SNPs were retained when (1) only two bases segregated in the panel, (2) both parental strains SS and T7 had read data for the site, (3) parental strains were both homozygous (defined as having a sample frequency of at least 0.9 for the most common base in each strain), (4) parents were fixed for different alleles, (5) at least 48 recombinant individuals yielded a genotype call, and (6) the frequency of heterozygous calls for the SNP was at most 75%. Individual genotype calls based on >500 reads were ignored when assessing SNP quality.

The number of SNPs surviving all filters was 571,937. Given low sequencing coverage, confidence in the calls for individual SNPs is low. Since F_13_ recombinant animals are expected to have long contiguous stretches of the genome with the same genotype (homozygous for either SS or T7, or heterozygous), we summed the SS and T7 allele read counts for the set of filtered SNPs present within non-overlapping blocks of 250 kb throughout the genome (S1 Table). We then converted the window-based read counts for each individual into called genotypes (i.e., SS/SS, SS/T7, or T7/T7) following a series of rules; (a) To call a genotype the minimum number of reads for the window must be 20 for autosomal windows or 10 for windows on the X chromosome (applied because of the difference in dosage between the X chromosome and the autosomes in males), (b) to call a homozygote, the number of SS reads must be at least 10 times greater than the number of T7 reads, or *vice versa*, and (c) to call a heterozygote the frequency of the T7 allele must be 0.3–0.7. Subsequently, we eliminated recombinant animals from the dataset if less than 40% of the window markers yielded a called genotype. We also masked entire genotype windows from the dataset if (i) fewer than 90% of the individuals were called for the window, and (ii) the minor allele frequency for the window was less than 5%. As a result, 426 markers were used for QTL mapping (X = 70, 2L = 86, 2R = 78, 3L = 87, 3R = 105; S2 Fig).

### QTL mapping

Following genotype filtering, 181 phenotyped F_13_ male recombinants remained. For those animals where both lobes were successfully measured (58%) we randomly selected one of the two lobes for QTL mapping. Both map estimation and QTL mapping were carried out within r/qtl [38]. Mapping was carried out using multiple imputation [39], stepping through the genome in 1 cM increments, and statistical significance was determined via 1000 permutations [40], estimating thresholds for the X and autosomes separately [41].

Confidence intervals on true QTL genetic locations were defined by a 2-LOD drop from each peak. We know the physical positions (in bp) of the markers, and their genetic positions (in cM) are estimated in r/qtl, therefore we can convert all genetic positions along the map to physical positions by virtue of data for the flanking markers. In turn, these physical positions were converted to cytological locations using the map conversion files available on FlyBase [25].

## Results and Discussion

### Recombinant genotyping

We queried over half a million SNPs in an F_13_ recombinant mapping panel derived from a pair of inbred strains of *D*. *melanogaster* (SS and T7), using low-pass whole genome sequencing for half the animals, and reduced-representation genotyping-by-sequencing [34] for the other half (S2 Table). Given the relatively low read coverage at each variant in each individual, and the mosaic haplotype structure of the recombinants, we elected to bin read data for neighboring SNPs in a set of 250 kb non-overlapping windows across the genome (see Materials and Methods). In contrast to individual SNP calls these consensus window genotype marker have high information content, and give an accurate picture of the genotypes of the recombinants (S2 Fig).

We tested whether the window-based genotypes were influenced by the genotyping method employed (MSG or WGS). We used the genomewide genotypes to generate a dissimilarity matrix for all pairs of recombinants, and using hierarchical clustering ('hclust' function in R) found no evidence that samples cluster based on the method used to obtain genotypes (S3 Fig). Thus, either genotyping method appears to work equivalently for the purposes of providing markers for the type of two-parent QTL mapping study we carry out here.

One concern with our window-based genotyping approach relates to those 250 kb windows for a given individual that contain a crossover. In such cases the genotype call will depend on the true position of the crossover, and the information available in the reads covering variable sites within the window. The crossover-containing window can receive the same genotype as that of the window immediately up- or downstream, either way slightly mis-estimating the true crossover position. Alternatively, if the mixture of genotypes at the SNPs up- and downstream of the crossover event within the window lead to read counts that fail to meet our thresholds (see Materials and Methods), the window will receive a no call. Examination of the genotypes of the recombinants (S2 Fig) suggests that in many cases a transition between segments having a different genotype is frequently accompanied by an intervening window lacking a genotype call, suggesting window markers harboring a crossover may often not receive a genotype. Methods using hidden Markov models (HMMs) have been implemented to directly use the low information SNP calls to estimate the genotype structure of recombinants (for example [34]). We anticipate an HMM method would yield similar estimates of crossover positions to our binning approach for the current population since it contains modest numbers of crossover events. HMM-based methods may be more beneficial with a highly recombinant population where intervals between adjacent crossovers are physically small.

We chose to generate an F_13_ advanced intercross mapping population in order to achieve enhanced QTL mapping resolution over an F_2_ design [42, 43]. We did not attempt to control breeding through the generations, and as such both selection and drift are likely to have played a role in shifting allele frequencies away from the expected ratio (50:50 SS:T7), as has been seen in previous advanced intercross populations of *Drosophila* [23, 30]. S4 Fig highlights three principal regions where allele frequency has skewed towards high T7 (right, telomeric end of 2R; left, telomeric end of X) or high SS (right, telomeric end of 3R). Regardless of the mechanism responsible for such allele frequency skew, it is difficult to identify QTL in regions dominated by a single allele, and indeed we ignored all those markers present in the 3.5 Mb region at the tip of the X for mapping, since all F_13_ recombinants were homozygous for T7 in this region. Since the posterior lobe is itself under sexual selection [2, 3, 8], selection could conceivably have acted through the trait of interest, making some lobe QTL more difficult to identify. Controlled breeding using random, single pair crosses, and ensuring parents contribute equal numbers of progeny to subsequent generations may lessen the allele frequency variation we observe [44], and minimize the action of certain selective forces (e.g., pre-copulatory sexual selection). However, relatively large numbers of crosses are needed to avoid the effects of drift, and given the trivial nature of uncontrolled fly breeding the additional work may simply not be practical in most cases.

### Genetic map

The standard *D*. *melanogaster* genetic map is 276 cM [25], while the map in our F_13_ population is 822 cM, an overall expansion of around 3X. This is approximately in line with theoretical expectations [45, 46]; For a two-parent advanced intercross F_*s*_ population, autosomal map expansion is *s*/2. Given that crossing over in flies occurs only in the female germline, *s* is equal to half the number of generations the population has experienced. Thus, map expansion for autosomes in an F_13_ panel is expected to be *s*/2 = (13/2)/2 = 3.3. On the X chromosome expected expansion is 2/3 of this value, or 2.2. Generally consistent with these values, observed map expansion was 2.8, 2.8, and 3.3 on chromosomes X, 2, and 3, respectively. Further increasing the number of generations of intercrossing would clearly provide additional gains in mapping resolution, but potentially at a cost of even greater allele frequency skew (for instance [30]).

### Phenotypic variation among genotypes

Previous surveys of posterior lobe variation within *D*. *melanogaster* have revealed abundant size and shape variation [23, 24], although the variation observed is substantially less pronounced than is seen across the four species of the *melanogaster* complex [4, 5], where male lobe shape is a principal marker of species identity. The two inbred strains selected for the current study—SS and T7—have different lobe morphologies (Fig 1); T7 individuals have narrower lobes than SS individuals, and the posterior section of T7 lobes (opposite the "point") is more curved than in SS lobes.

**Fig 2:**
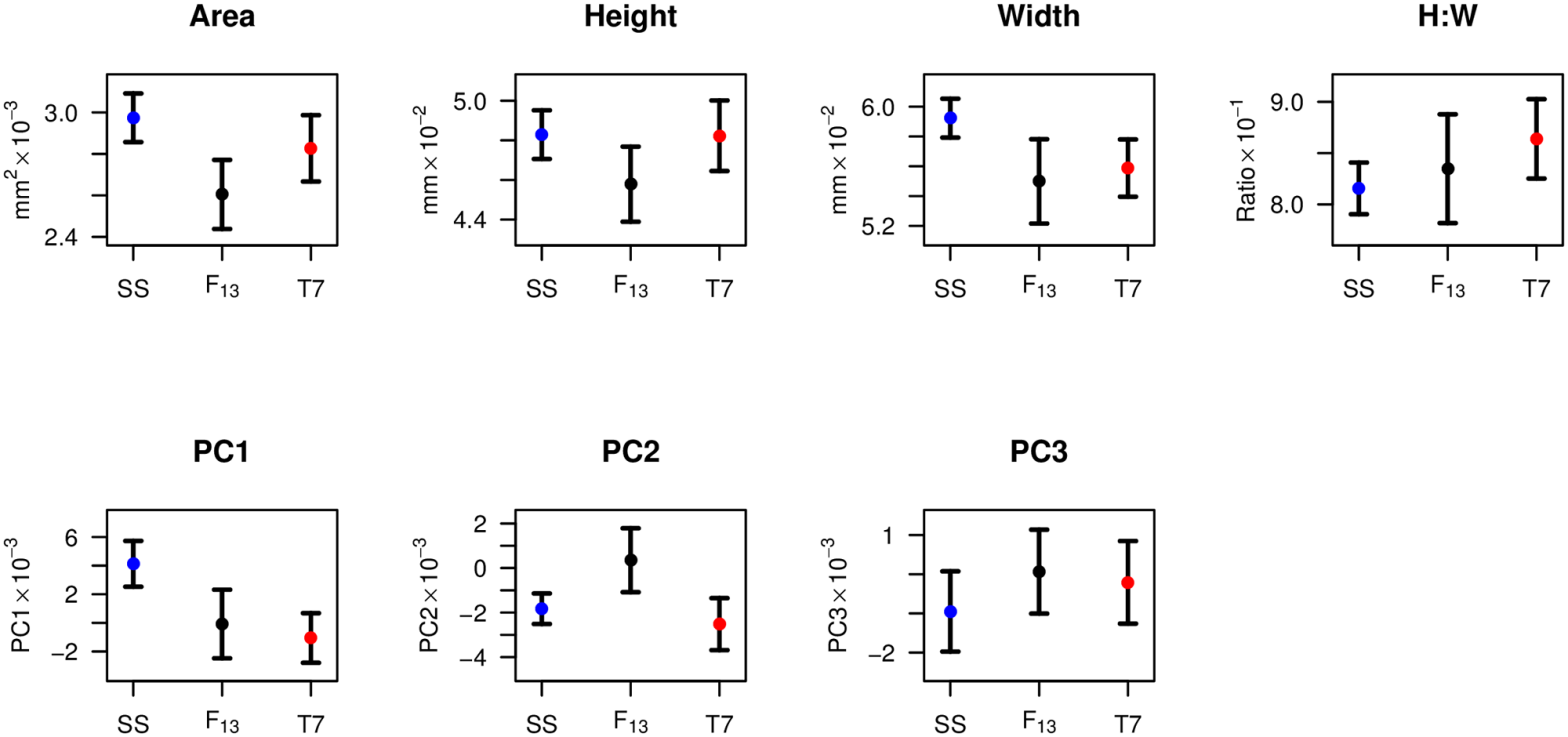
Summary of posterior lobe size and shape phenotypes in parental strains and recombinants. Points represent the mean phenotype across lobes for each class, and whiskers represent 1-SD. Sample sizes are 14, 303, and 32 lobes for SS, recombinants, and T7, respectively. The total number of independent individuals scored was 8, 192, and 21.

Since among-individual morphological variation for complex structures such as the posterior lobe is not completely captured by simple descriptors (e.g., width), we additionally used a combination of elliptic Fourier analysis and PCA to describe variation across all lobes in the study (i.e., SS, T7, and F_13_) in terms of principal components (PCs). The top three PCs collectively explain nearly 90% of the morphological variation in the dataset (PC1 = 54.6%, PC2 = 24.4%, PC3 = 10.5%). Fig 2 highlights the differences among the two parental strains, and the recombinant animals considered as a single class, for three measures of lobe size (area, height, width), a simple measure of lobe shape (ratio of height to width, or H:W), and the first three PCs. The parental strains can be discriminated by area (Welch's *t*-test, *p* = 0.001), width (*p* < 10^−7^), H:W (*p* < 10^−4^), PC1 (*p* < 10^−9^), PC2 (*p* = 0.02), and PC3 (*p* = 0.03), but not by height (*p* = 0.9). For area, height, and PC2 the set of recombinant F_13_ animals have phenotypes that, on average, fall outside the range of the parental strains, potentially indicative of transgressive segregation for these phenotypes. We note that any lobe size (area, height, width) variation is not necessarily a result of overall body size variation, since previous investigators have found that measures of body size are not predictive of lobe size [16–18, 47].

Since the first three PCs explain much of the shape variation among individuals in this study, and PC1 is highly significantly different between the parent strains used for mapping (Fig 2), we sought to define the aspects of lobe phenotype the PCs describe. First, we used a single lobe from each of the F_13_ individuals to carry out correlations among phenotypes (Table 1). PC1 is strongly positively correlated with lobe width (Pearson's *r* = 0.59, *p* < 10^−10^), strongly negatively correlated with H:W (*r* = –0.58, *p* < 10^−10^), and has a very minor negative correlation with lobe height (*r* = –0.16, *p* = 0.03). This implies that the relatively simplistic H:W ratio describes a substantial fraction of the shape variation among lobes (see also [23]), with much of the PC1 variation being effectively described as changes in lobe width. Nonetheless, inspection of variation among lobes ordered by PC1 suggests that other phenotypes are changing along with width and H:W (S5 Fig), although it is not particularly clear how to describe these additional shape changes. This underlies the benefit and the cost of using EFA-PCA to describe shape changes for outlines lacking clear landmarks; One can capture variation that is difficult to define, but one lacks a clear definition that may be helpful to understand the phenotype. PC2 is tightly negatively correlated with lobe area (*r* = –0.89), and PC2 can be considered a proxy for overall lobe size (Table 1). It is not clear what feature of lobe morphology PC3 explains, and given the limited contribution this measure makes to overall lobe variation (10.5%), we do not focus on this PC further.

**Table 1:** Correlations among posterior lobe size and shape phenotypes.

	Area	Height	Width	H:W	PC1	PC2	PC3
Area	—	0.69	0.73	–0.16	0.31	–0.89	–0.08
Height	***	—	0.07	0.58	–0.16	–0.60	–0.54
Width	***	ns	—	–0.77	0.59	–0.64	0.40
H:W	*	***	***	—	–0.58	0.15	–0.67
PC1	**	*	***	***	—	–0.02	0.01
PC2	***	***	***	*	ns	—	–0.17
PC3	ns	***	**	***	ns	*	—

Correlations among traits (Pearson's *r*) above the diagonal, and significance of the correlation tests below the diagonal (*** *p* < 10^−10^; ** *p* < 0.0001; * *p* < 0.05; ns, *p* > 0.05). PCs are orthogonal, and by definition correlations among them will be zero using the full dataset. However, only a single lobe from each individual is used for these tests, so non-zero correlations can be observed.

### QTL mapping of posterior lobe variation

Previous studies have detected loci contributing to intraspecific posterior lobe variation within *D*. *melanogaster* using both QTL mapping [23] and a GWAS [24] using 155 of the sequenced inbred strains of the *Drosophila* genetic reference panel, DGRP [48, 49]. The difference in power between linkage-based QTL studies and population-based association studies is well known. Loci mapped in QTL studies routinely explain large fractions of the heritable variation in the cross (for example [50, 51]), while association studies generally struggle to identify causative variants explaining anything more than a fraction of the heritability for the trait [52, 53]. It is also very clear that association approaches can have much greater mapping resolution than traditional F_2_ QTL mapping studies due to a radical difference in the number of crossover events in the history of the mapping panels. Here, we sought to use a linkage-based, advanced intercross mapping panel to benefit from the power of a linkage-based design, but garner greater mapping resolution by including more crossover events [42, 54], although obviously falling short of the resolution possible with a GWAS.

We note that one can increase crossover number, and thus QTL mapping resolution, by simply increasing the number of F_2_ (or backcross) individuals tested, rather than by generating an advanced generation population. However, for any given level of resolution desired, a larger sample size is required for a population that has undergone fewer generations of recombination [55]. Therefore, the appropriate mapping design needs to weigh the additional phenotyping burden of a very large F_2_ study against any difficulty generating an advanced intercross population.

**Fig 3:**
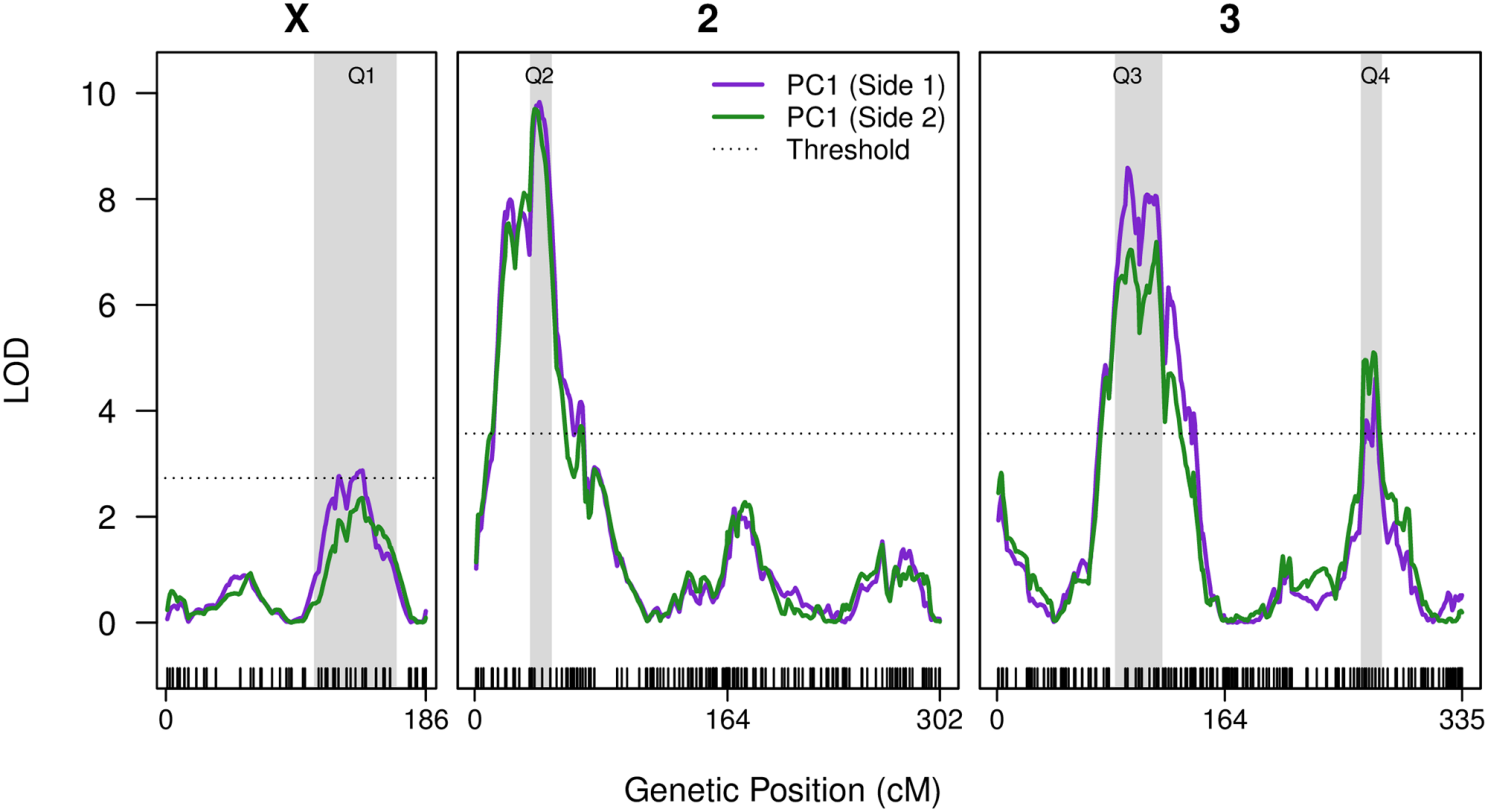
Four QTL mapped for PC1 lobe shape variation. The two solid curves present evidence for mapped QTL. The purple curve is derived from a dataset using one randomly-selected lobe from each of the recombinants were both lobes were successfully scored, and the only lobe present for the remaining animals. The green curve uses the other lobe for the 58% of animals where both were scored. The horizontal dotted lines are the permutation-derived, genomewide 5% statistical thresholds (X; LOD = 2.73, Autosomes; LOD = 3.57). The positions of the markers are presented as ticks along the inside of the x-axis. The 2-LOD drop support intervals for each QTL are highlighted as gray bars.

We mapped variation for three posterior lobe size traits (area, height, width) and four lobe shape traits (H:W, PC1, PC2, PC3) in the bi-parental F_13_ mapping population using r/qtl [38]. We provide the r/qtl input file as S3 Table and the LOD score output as S4 Table. Fig 3 shows QTL mapping results for the principal measure of morphology, PC1, for which we map four QTL, one on the X chromosome, and three on the autosomes. For those recombinant animals were we successfully scored both lobes (58%), only one of the two was initially used for mapping (Fig 3, purple curve). Nevertheless, we identify the same peaks regardless of which lobe side is chosen (Fig 3, compare purple and green curves), consistent with strong bilateral symmetry observed for this trait: The correlation between lobe sides for PC1 is *r* = 0.75 (*p* = 10^−10^). Using various routines in r/qtl [38] we explored the properties of the four PC1 QTL further. The additive model, *y* = Q1+Q2+Q3+Q4 explains 41.6% of the PC1 variation among the F_13_ individuals (using the 'fitqtl' function). Including all possible interaction terms in the model (i.e., *y* = Q1×Q2×Q3×Q4) increases the fraction of the variance explained to 54.5%, but allowing pairwise interactions among these four QTL did not significantly improve the fit of the model (using 'addint'). Additionally, the 'scantwo' function, which allows two-dimensional scans for multiple QTL, did not show clear evidence for interactions among the four PC1 loci. Finally, we tested whether there was evidence for additional QTL given the presence of Q1–Q4 using 'addqtl'. There was some suggestion of another QTL at the very tip of 3L (at 0 cM on chromosome 3, Fig 3), although evidence was not strong, and we do not consider this potential peak further.

**Fig 4:**
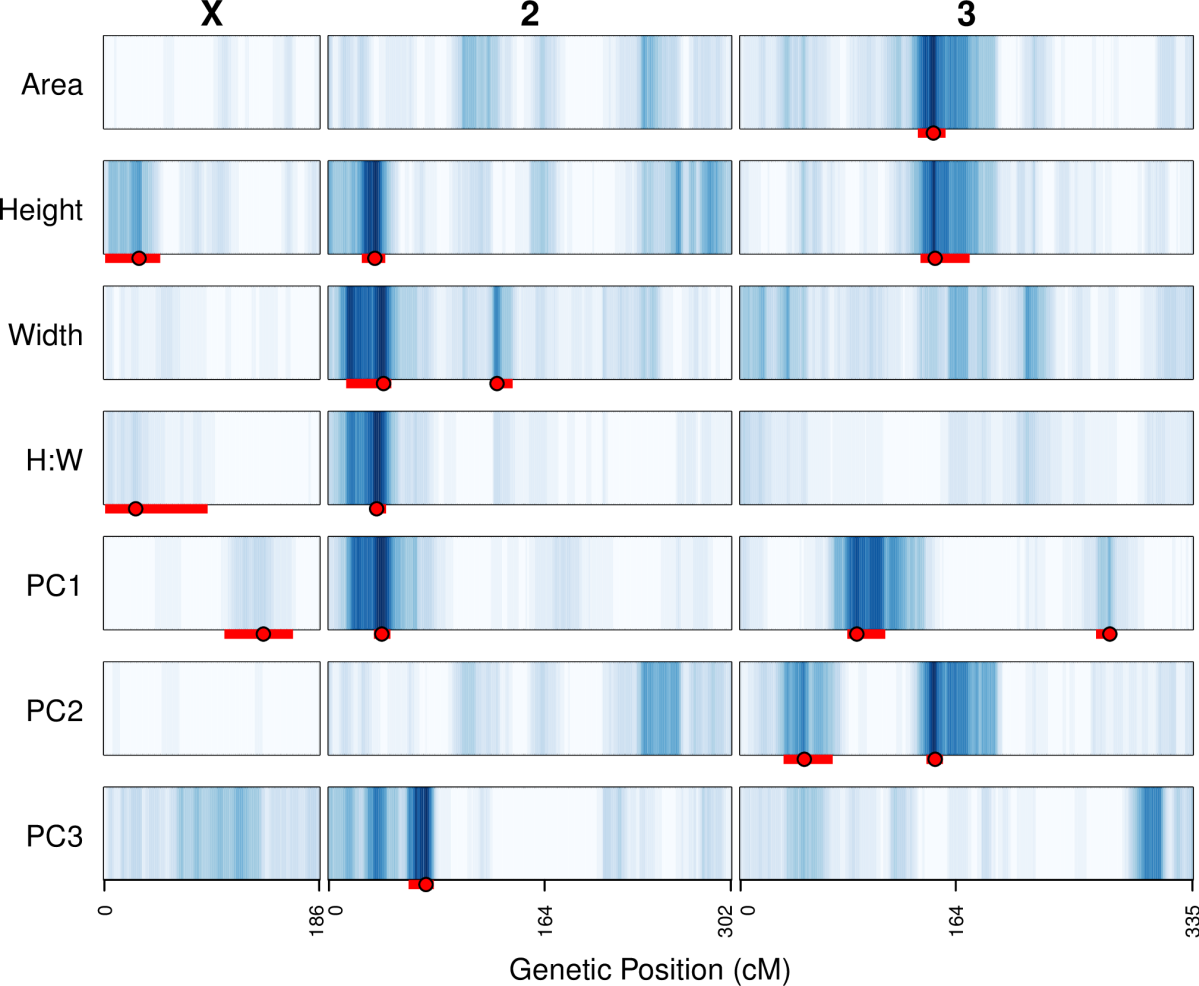
Positions of QTL mapped for all lobe shape and size traits. The LOD scores at each test position along the genome have been converted to blue-scale, with darker blue corresponding to higher LOD scores. LOD score color-encoding was carried out separately for each trait, such that the maximum LOD score for every trait was given the same dark blue color, facilitating comparisons across LOD curves with varying maxima. Intensities on the X chromosome are lower given the lower statistical thresholds for QTL detection on the X. The peak positions for each QTL are presented as solid red points, and 2-LOD drop intervals are shown as red bars.

We performed similar routines to examine the pair of QTL mapped to chromosome 3L that contribute to PC2 (Fig 4, second panel from the bottom), a measure of lobe morphological variation that is highly negatively correlated with lobe area. The model *y* = Q1+Q2 explains 25.2% of the variation in PC2, and no additional QTL or interaction terms improved the fit of the model.

Table 2 provides details of the four PC1 and two PC2 QTL, and S6 Fig shows the effect of each QTL genotype on phenotype in the F_13_ panel. All QTL explain appreciable fractions of the variation, with 4/6 explaining >10% of the variation in the traits (Table 2). Single, relatively large-effect loci causative variants may underlie these QTL, although given the size of the implicated intervals (Table 2), multiple linked variants may be responsible for each QTL (see [56]).

**Table 2:** Details of QTL mapped for PC1 and PC2 lobe morphology descriptors.

	PC1-Q1	PC1-Q2	PC1-Q3	PC1-Q4	PC2-Q1	PC2-Q2
Chromosome	X	2L	3L	3R	3L	3L
LOD score	2.9	9.8	8.6	4.6	4.3	6.0
Variance explained (%) ^a^	1.6	13.7	10.1	2.5	11.1	15.0
Additive effect ^a^	3.4	13.3	–10.9	1.4	–6.5	2.2
Dominance effect ^a^	—	–0.4	1.7	–7.4	1.4	–9.4
Interval (cM) ^b^	106–165	36–50	85–119	262–277	34–72	146–155
Physical position (Mb) ^b^	13.6–19.4	4.1–5.6	10.4–13.7	21.7–23.7	4.5–9.5	18.7–21.2
Cytology ^c^	12A-18C	24D-25F	67E-70C	93F-95A	64B-67B	75D-78C
Number of genes ^d^	619	172	377	251	580	331

^*a*^ The fraction of the phenotypic variance explained, and the additive and dominance effects, are derived from the r/qtl function 'fitqtl'. The effects have been multiplied by 10^4^ for clarity.

^*b*^ QTL intervals are defined by 2-LOD drops from the peaks. Genetic distance is given on the expanded map (map distances on the typical *D*. *melanogaster* genetic map will be approximately 3 times shorter.) Physical distance is provided relative to Release 6 of the genome.

^*c*^ Cytological positions were derived from the physical positions (in bp) using the conversion files on FlyBase [25].

^*d*^ Only the numbers of protein-coding genes are provided.

At PC1-Q1 and PC1-Q2 replacing a T7 allele with an SS allele increases the phenotype in a largely additive fashion (Table 2), making the lobe more SS-like (S5 Fig). PC1-Q3 shows the opposite effect; SS alleles decrease the phenotype, making the lobe less parental SS-like. Since the parental inbred lines were not directionally-selected for lobe morphology, there is no expectation QTL will act in a consistent direction. Finally, PC1-Q4 principally shows an overdominant effect (Table 2) with heterozygous genotypes having a lower phenotype compared to either homozygous genotypic class (S6 Fig). PC2-Q1 acts additively, while PC2-Q2 also shows an overdominant effect (Table 2; S6 Fig). The combination of additive and dominant QTL action is consistent with our previous QTL mapping work employing an independent pair of starting parental strains [23].

We also obtained genotypes for window markers across the small fourth chromosome. For each individual, all genotype calls at windows across the chromosome were consistent (S1 Table), allowing us to generate a single, consensus chromosome 4 genotype for each recombinant. For those 144 recombinants with a called genotype we carried out linear regressions of genotype on phenotype for each of the seven lobe traits using the 'lm' function in R. In no case was there an association between fourth chromosome genotype and phenotype (*p* > 0.53 in all cases).

### Overlap of QTL describing different lobe shape/size metrics

Fig 4 depicts the positions of QTL mapped for all seven measures of posterior lobe size and shape (see also S5 Table). If we consider QTL to coincide when the 2-LOD drop intervals overlap we identify a total of nine independent QTL, although without considerable further work we cannot be completely confident the underlying genetic basis of overlapping QTL is the same. The largest effect PC1 QTL on 2L, PC1-Q2 is located in the same position as QTL mapped for lobe height, width, and H:W, suggesting this locus has robust effects on several metrics of lobe morphology. Neither of the other three PC1 QTL overlap with QTL found for other traits, suggesting they confer effects on aspects of lobe shape that are not easily defined with simple measures of trait morphology. The major PC2 QTL, PC2-Q2 overlaps with QTL for area and height (Fig 4), consistent with the strong correlation between PC2 and area (Table 1). The other PC2 QTL, PC2-Q1 and the sole PC3 QTL do not overlap any loci implicated for other aspects of lobe morphological variation, again suggesting they are responsible for morphological variation that is difficult to describe without PCs. Generating introgression lines, specifically isolating QTL alleles from each parent in the genetic background of the other, would be a fruitful approach to help define the morphological change conferred by the QTL, while additionally helping resolve the causative variants.

### Overlap of posterior lobe loci among studies

The advanced intercross design of our mapping population succeeded in mapping QTL to smaller regions than is possible with an F_2_ mapping population with a modest sample size (e.g., [23]). On the unexpanded *D*. *melanogaster* genetic map PC1 and PC2 QTL are mapped to 2–18 cM, corresponding to physical intervals of 1.5–5.8 Mb (Table 2). Nonetheless, these QTL intervals still implicate fairly large numbers of protein-coding genes (Table 2 and S6 Table). Since other work has mapped loci contributing to posterior lobe variation within *D*. *melanogaster* [23, 24], and studies have used expression analysis or RNAi to define plausible candidate posterior lobe genes [19, 27, 28], we sought to investigate any overlap among QTL and functional candidates in *D*. *melanogaster*. See S1 Data for a summary of intervals, genes, and sites previously implicated in posterior lobe development or variation in *D*. *melanogaster*.

McNeil at al. [23] mapped three autosomal QTL for a measure of posterior lobe shape defined by EFA-PCA in an F_17_ cross between the inbred lines *Samarkand ry*^*506*^ and b3852, and two of these QTL coincide with regions mapped in the present study. The QTL mapped to cytological location 66B–69B by McNeil at al. [23] overlaps with both PC1-Q3 and PC2-Q1 mapped here (Table 2). Given that PC axes are defined relative to a specific dataset, there is no necessity that the shape/size metric represented by a given PC is consistent across studies. The observation that two independent QTL intervals overlap the larger interval implicated in McNeil et al. [23] could imply multiple loci underlie this previously-mapped QTL. In addition, the QTL mapped to 75F–86C by McNeil et al. [23] overlaps PC2-Q2 identified here, although PC2-Q2 implicates a much smaller region of the genome (2.5 Mb versus 12.0 Mb, Table 2). Under the assumption the underlying genetic basis of these loci is the same, combining the two QTL studies can help to resolve the genes (S6 Table) underlying phenotypic variation in the two different crosses.

GWAS designs can provide additional mapping resolution over linkage-based studies. In flies, where linkage disequilibrium extends over relatively short distances [49, 57], a significantly associated site would be expected to reside very close to the actual causative variant (and potentially *be* the causative variant). A GWAS for posterior lobe morphology found no nucleotide variants formally associated with any of the tested lobe shape/size phenotypes after correction for multiple tests [24], a result of the lack of power to find modest effect causative loci in the DGRP design given the small number of genotypes and the severe correction for multiple tests that must be applied [52]. However, a number of nominally significant sites were identified, and as a class one might anticipate this group to be enriched for variants truly associated with lobe variation. Although the actual level of enrichment of true positives is unknown, an appealing possibility is that one could combine high-powered linkage-based approaches to broadly map QTL, and low-powered, but high-resolution population-based association approaches to fine map within QTL intervals, thereby reducing the burden of multiple testing. Thus, we examined whether any of the nominally-associated loci implicated by Takahara & Takahashi [24] are present within our QTL intervals. Nine genes within the six PC1/PC2 we map in this study (Table 2) additionally contain GWAS variants with *p*-values below 10^−5^ (S1 Data); *hang* (PC1-Q1), *Hel25E* (PC1-Q2), *bru-3*, *CAH2*, *CG43693*, and *Nrx-IV* (PC1-Q3), *Sec13* (PC1-Q4), *GluRIB* and *unc-13-4A* (PC2-Q1). Evidence for the role of these genes in genital morphology or development is currently lacking [25]. However, specific validation of these genes using quantitative complementation tests [58, 59], reciprocal hemizygosity tests [60], or CRISPR (swapping putatively-functional alternative alleles into a common genetic background), may be able to identify candidate genes contributing to lobe morphological variation. Simultaneously, such tests may help validate whether using a combination of QTL and association mapping can aid in the dissection of complex phenotypes to the nucleotide level.

A number of studies have used functional tests or expression measurements in the developing male genitalia to identify plausible candidate genes contributing to lobe morphogenesis and/or adult morphology. Chatterjee et al. [27] identified a set of 22 genes showing consistent differences between male and female larval genital discs, the primordia of the adult genitalia. Three of these genes—*toe*, *eyg*, and *caup*—are within the PC1-Q3 interval (Fig 3 and Table 2), suggesting—as we have previously [23]—that genes with sex-based gene expression in the developing genitalia may be plausible candidates to harbor functional variation contributing to lobe morphology. Glassford et al. [28] provide a number of genes involved in lobe development. Two of these are present within QTL mapped here; *upd1*, a gene found to be expressed in the developing lobe (figure four in [28]) is within PC1-Q1, and *RhoGEF64C*, that when knocked down specifically in the developing male genitalia leads to reduced lobe size (figure four in [28]), resides within PC2-Q1. Finally, Tanaka et al. [19] employed RNAi targeted to wing, leg, and genital discs in *D*. *melanogaster* to test the effects of multiple genes implicated in the differences in lobe morphology between *D*. *simulans* and *D*. *mauritiana*. All these genes are located in short introgressed regions that have significant effects on morphological differentiation between species in either the morphology of the lobe or number of bristles on the clasper (another genital structure critical for mating success). A number of genes with significant RNAi effects reside within QTL we map in the present study; *CG11652*, *CG14130*, *CG32081*, *CG32082*, *Mob2*, and *wls* (PC1-Q3), *CG6673*, *CG14835*, *CG14838*, *dally*, *Mcm7*, *msl-3*, *Prm*, and *Surf1* (PC2-Q1). Of particular interest with respect to this gene set is the potential for it to contain one or more loci that harbor allelic variation contributing to both intra- and interspecific differences in posterior lobe morphology.

## Conclusion

Dissecting the genetic basis of intraspecific variation in the morphology of genital structures that exhibit rapid evolution among species can provide information on the loci that are the targets of sexual selection. We used a pair of genetically and phenotypically distinct inbred lines of *D*. *melanogaster* to generate an advanced intercross population enabling powerful, and relatively high resolution genetic mapping of variation in the posterior lobe, a structure that is essential for mating in the species. By making use of genotyping-by-sequencing technologies, and a fairly simple analytical pipeline employing standard genomics software (e.g., bwa, GATK), we were able to efficiently collect genomewide genotypes for a set of highly-recombinant individuals. We identified several, modest-effect loci influencing lobe morphology, some of which overlap with previously-identified QTL, and with candidates from functional genetics studies. We are obviously some way from our final goal of identifying the precise series of naturally-segregating causative variants contributing to posterior lobe variation. Nonetheless, by combining disparate datasets we can home in on likely candidates, paving the way for functional validation of causative genes, and testing for the action of selective forces at these loci.

## Supporting Information

S1 Data Summary of previously identified loci. Information about QTL and loci previously implicated in the genetic control of posterior lobe development and adult morphology in *Drosophila melanogaster*.
(PDF)

S1 Fig Posterior lobe phenotype acquisition workflow. (PDF)

S2 Fig Genotypes of recombinant individuals. Each of the recombinant individuals (rows) is given a genotype for a set of non-overlapping 250 kb windows along the genome (columns). Windows where no genotype call was made are shown in white. The top two rows of the image represent the homozygous parental T7 and SS parental strains, which receive homozygous calls (red and blue, respectively) for all windows genotyped. The thick black line at the bottom of the image depicts which genotyped windows were used as markers for QTL mapping.
(PDF)

S3 Fig Clustering recombinants based on their genotype. The number of differences in genotype was calculated between every pair of recombinants for the set of window-based markers on the five major chromosome arms (X, 2L, 2R, 3L, 3R). This dissimilarity, or mismatch matrix was then subjected to hierarchical clustering using the 'hclust' function in R (GenoTree <- hclust(as.dist(MismatchMatrix),method = "average")). The plot depicts the resulting dendrogram, with recombinants highlighted based on genotyping data collection method (black, MSG; red, WGS). There is no clear clustering of animals genotyped with the same method.
(PDF)

S4 Fig Parental allele frequencies in the recombinant mapping population. Each point depicts the frequency of the T7 allele in the panel of 181 recombinant individuals at one of the 250kb markers. Windows with T7 allele frequency above 0.8 or below 0.2 are highlighted in red.
(PDF)

S5 Fig Subset of posterior lobes sorted by the value of PC1 shape phenotype. Four lobes from each parental strain are presented (SS, blue; T7, red), along with 88 lobes from recombinant individuals (black). The number within each image is the PC1 (× 10^4^) value assigned to the lobe.
(PDF)

S6 Fig Effect plots for all six PC1 and PC2 QTL. The phenotype data for the recombinants is plotted against their genotypes at the six QTL peaks. For each genotypic class, the mean (+/– 1-SE) phenotype is also presented.
(PDF)

S1 Table Window-based read counts. For each individual (see "ID" column) genotyped by either MSG or low-pass WGS (see "GenotypeMethod" column) the sum of the number of reads consistent with either the T7 ("T7reads") or SS ("SSreads") allele at all SNPs within a series of non-overlapping windows of 250 kb throughout the genome is presented. Thus, each window is presented as a pair of columns.
(TXT)

S2 Table Genotyping method for each recombinant. A list of the sample IDs for all 181 recombinants, along with a column stating whether the individual was genotyped with low pass whole genome sequencing (WGS) or reduced representation multiplexed shotgun genotyping (MSG).
(TXT)

S3 Table Input file for QTL mapping. This file can be directly read into r/qtl using 'read.cross'. Recombinant individuals are defined using an internal lab ID. Markers are given genetic distances calculated within r/qtl. Genotypes are given using the following format: A = T7 homozygote, H = heterozygote, B = SS homozygote, NA = no call.
(CSV)

S4 Table LOD scores from QTL mapping. The "Name" column is the name of the test position, which is either a marker (format similar to "Chr2L.1") or an intermarker position (format similar to "c2.loc1"), "Chr" is the chromosome, and "Position_cM" is the genetic position of the marker estimated via r/qtl. Columns "PositionR6_Lower_Mb" and "PositionR6_Upper_Mb" give the upper and lower boundaries of the 250kb marker genotype windows, and the equivalent interpolated boundaries for intermarker positions. The "*.LOD" columns give the LOD associated with hypothesis tests for the presence of a QTL for each of the seven lobe shape/size traits.
(TXT)

S5 Table Positions of QTL mapped for all 7 traits. (PDF)

S6 Table Protein coding genes present within PC1 and PC2 QTL intervals. The "Phenotype" column is the trait the QTL is mapped for, the "QTL" column is the name of the QTL (corresponding to the names in Table 2), the "Cytology" and "PhysicalPos_R6" columns are the cytological locations and the physical positions (in genome Release 6 coordinates) of the genes, respectively, and "GeneSymbol" and "FBgn" provide information on the gene names.
(TXT)
