# Supplementary material for: Mapping QTL Contributing to Variation in Posterior Lobe Morphology between Strains of Drosophila melanogaster
Source: PLoS One. 2016 Sep 8;11(9):e0162573. doi: 10.1371/journal.pone.0162573 (PMC5015897; doi:10.1371/journal.pone.0162573)
Supplement: S1 Data — Information about QTL and loci previously implicated in the genetic control of posterior lobe development and adult morphology in Drosophila melanogaster. (PDF) [file pone.0162573.s001.pdf]

Supplementary Data S1. Summary of QTL and loci previously implicated in the genetic control of posterior lobe development and adult morphology in *Drosophila melanogaster.*

McNeil et al. (2011) - PMID: 22384345. See Table 3.

Identified three QTL for a measure of posterior lobe shape (PC1):

QTL	*Chr.*	*Cytology*
Q1	2L	27E-29A
Q2	2L	66B–69B
Q3	3c	75F–86C

Takahara & Takahashi (2015) - PMID: 26182199. See Supplementary Tables 2, 3, and 4.

No sites were significant after formal correction for multiple tests, but a number of variants showed associations with measures of posterior lobe shape (PC1, PC2) and size at a nominal significance threshold of $p < 10^{-5}$.

PC1 (16 variants at $p < 10^{-5}$)

Variant ID [a]	*p*-value [b]	Position (Release 6) [c]	Gene [d]
2L_11917838_SNP	8.7×10^{-6}	2L:11917838	*Pde1c* (FBgn0264815)
2L_16982042_SNP	3.5×10^{-6}	2L:16982042	*beat-IIIb* (FBgn0053179), *CG44406* (FBgn0265579)
2L_17282029_SNP	9.3×10^{-6}	2L:17282029	-
2L_582234_SNP	4.7×10^{-6}	2L:582234	-
2R_16809817_INS	7.0×10^{-6}	2R:20922312	*Rx* (FBgn0020617)
2R_17146075_SNP	8.4×10^{-6}	2R:21258570	*Rgk3* (FBgn0085426)
2R_8919718_SNP	2.9×10^{-6}	2R:13032213	-
3L_15296932_SNP	4.8×10^{-6}	3L:15303832	-
3L_15297000_SNP	5.2×10^{-6}	3L:15303900	-
3L_15297065_SNP	6.6×10^{-7}	3L:15303965	-
3L_16826245_SNP	1.3×10^{-6}	3L:16833145	-
3L_9276471_SNP	1.1×10^{-6}	3L:9283371	*GluRIB* (FBgn0264000)
3R_15744495_SNP	5.8×10^{-6}	3R:19918773	*CG4770* (FBgn0038751)
3R_19183951_SNP	4.6×10^{-6}	3R:23358229	*Sec13* (FBgn0024509)
3R_19184076_SNP	2.9×10^{-7}	3R:23358354	*Sec13* (FBgn0024509)
X_20429265_INS	8.9×10^{-6}	X:20558238	*RunxB* (FBgn0259162)

PC2 (14 variants at $p < 10^{-5}$)

Variant ID [a]	*p*-value [b]	Position (Release 6) [c]	Gene [d]
2L_16460631_SNP	1.0×10^{-5}	2L:16460631	-
2L_5540863_SNP	1.8×10^{-6}	2L:5540863	*Hel25E* (FBgn0014189)
2R_18594569_SNP	4.0×10^{-6}	2R:22707064	*Obp58d* (FBgn0034770)
2R_18659021_SNP	9.9×10^{-6}	2R:22771516	*CG42260* (FBgn0259145)
2R_18914361_SNP	2.9×10^{-6}	2R:23026838	*twi* (FBgn0003900), *CG42741* (FBgn0261705)
2R_7583208_SNP	1.0×10^{-5}	2R:11695703	*CG13197* (FBgn0062449)
3L_7290732_SNP	5.5×10^{-7}	3L:7297632	*unc-13-4A* (FBgn0035756)
3R_10150581_SNP	3.8×10^{-7}	3R:14324859	*Afti* (FBgn0038223)
3R_11532259_SNP	3.5×10^{-6}	3R:15706537	*CG5404* (FBgn0038354)
3R_11532260_SNP	1.5×10^{-6}	3R:15706538	*CG5404* (FBgn0038354)
3R_11532266_MNP	3.3×10^{-6}	3R:15706544	*CG5404* (FBgn0038354)
3R_11532271_SNP	2.5×10^{-6}	3R:15706549	*CG5404* (FBgn0038354)
3R_2137747_SNP	4.3×10^{-6}	3R:6312025	*Osi17* (FBgn0037427)
X_16331129_DEL	6.2×10^{-6}	X:16437096	*hang* (FBgn0026575)

Size (15 variants at $p < 10^{-5}$)

Variant ID [a]	*p*-value [b]	Position (Release 6) [c]	Gene [d]
2L_1823396_SNP	4.2×10^{-6}	2L:1823396	-
2L_7045893_SNP	8.5×10^{-6}	2L:7045893	*milt* (FBgn0262872)
2R_13646097_DEL	4.8×10^{-6}	2R:17758592	*HLH54F* (FBgn0022740)
2R_13815024_SNP	9.1×10^{-6}	2R:17927519	-
2R_13815048_SNP	2.8×10^{-6}	2R:17927543	-
3L_11031740_INS	5.3×10^{-6}	3L:11038640	*CG43693* (FBgn0263776)
3L_11031783_SNP	8.4×10^{-6}	3L:11038683	*CG43693* (FBgn0263776)
3L_11037323_DEL	4.9×10^{-6}	3L:11044223	*CG43693* (FBgn0263776)
3L_11037405_SNP	2.3×10^{-6}	3L:11044305	*CG43693* (FBgn0263776)
3L_12148634_SNP	2.5×10^{-6}	3L:12155534	*Nrx-IV* (FBgn0013997)
3L_12175730_SNP	2.4×10^{-7}	3L:12182630	*CAH2* (FBgn0027843)
3L_13764854_SNP	8.7×10^{-6}	3L:13771754	*bru-3* (FBgn0264001)
3L_13764855_SNP	8.3×10^{-6}	3L:13771755	*bru-3* (FBgn0264001)
3L_20214515_INS	8.0×10^{-6}	3L:20221415	-
3R_14913820_SNP	3.3×10^{-6}	3R:19088098	*cdi* (FBgn0004876)

[a] Variant ID from DGRP analysis software (dgrp2.gnets.ncsu.edu/). The first component is the chromosome on which the variant resides, the second is the position in Release 5 of the *D. melanogaster* reference genome, and the third is the type of variant (SNP, INS, DEL).

[b] The *p*-value of the association test.

[c] The position of the variant on Release 6 of the reference genome (converted from Release 5 positions via flybase.org/static_pages/downloads/COORD.html).

[d] The name (and FlyBase ID) of any gene in which the variant resides. A "-" implies the variant does not reside in a gene.

Chatterjee et al. (2011) - PMID: 21343364.

Found 23 genes with male- or female-biased gene expression in the genital disc across three developmental timepoints. (Two of these genes - *CG4267* and *CG31686* - have since been merged into the single gene *CG4267*.)

Chr.	*Gene Name*	*FlyBase ID*
X	*CG4766*	FBgn0027546
X	*Nep1*	FBgn0029843
X	*lz*	FBgn0002576
X	*FucTC*	FBgn0044872
2	*al*	FBgn0000061
2	*CG4267*	FBgn0264979
2	*CG31686*	FBgn0264979
2	*msl-2*	FBgn0005616
2	*salr*	FBgn0000287
2	*ap*	FBgn0267978
2	*Wnt2*	FBgn0004360
2	*Poxn*	FBgn0003130
3	*bab1*	FBgn0004870
3	*toe*	FBgn0036285
3	*eyg*	FBgn0000625
3	*caup*	FBgn0015919
3	*AP-2*	FBgn0261953
3	*dsx*	FBgn0000504
3	*Ctr1B*	FBgn0062412
3	*abd-A*	FBgn0000014
3	*bnl*	FBgn0014135
3	*Dr*	FBgn0000492

Glassford et al. (2015) - PMID: 26343453.

Highlighted a number of genes expressed in the developing genital disc, and those having effects on the posterior lobe following RNAi.

Chr.	*Gene Name*	*FlyBase ID*
X	*dome*	FBgn0043903
X	*hop*	FBgn0004864
X	*upd1*	FBgn0004956
2L	*eya*	FBgn0000320
2L	*salm*	FBgn0261648
2R	*en*	FBgn0000577
2R	*Poxn*	FBgn0003130
3L	*RhoGEF64C*	FBgn0035574
3R	*Abd-B*	FBgn0000015
3R	*Cad86C*	FBgn0261053
3R	*crb*	FBgn0259685
3R	*ems*	FBgn0000576
3R	*Stat92E*	FBgn0016917

Tanaka et al. (2015) - PMID: 25783699. See Figure 4.

Identified several genes that influence posterior lobe morphology in *D. melanogaster* via RNAi. These candidates were selected based on their presence within regions of *D. mauritiana* that when introgressed into a *D. simulans* background affect genital morphology.

Chr.	*Gene Name*	*FlyBase ID*
3	*CG6673*	FBgn0035906
3	*CG11652*	FBgn0036194
3	*CG14130*	FBgn0036210
3	*CG14835*	FBgn0035782
3	*CG14838*	FBgn0035799
3	*CG32055*	FBgn0052055
3	*CG32081*	FBgn0052081
3	*CG32082*	FBgn0052082
3	*dally*	FBgn0263930
3	*iPLA2-VIA*	FBgn0036053
3	*Mcm7*	FBgn0020633
3	*Mob2*	FBgn0259481
3	*msl-3*	FBgn0002775
3	*Prm*	FBgn0003149
3	*Rac1*	FBgn0010333
3	*Surf1*	FBgn0029117
3	*wls*	FBgn0036141
