# Supplementary material for: Mapping QTL Contributing to Variation in Posterior Lobe Morphology between Strains of Drosophila melanogaster
Source: PLoS One. 2016 Sep 8;11(9):e0162573. doi: 10.1371/journal.pone.0162573 (PMC5015897; doi:10.1371/journal.pone.0162573)
Supplement: S1 Fig — (PDF) [file pone.0162573.s002.pdf]

**Supplementary Figure S1.** Posterior lobe phenotype acquisition workflow.

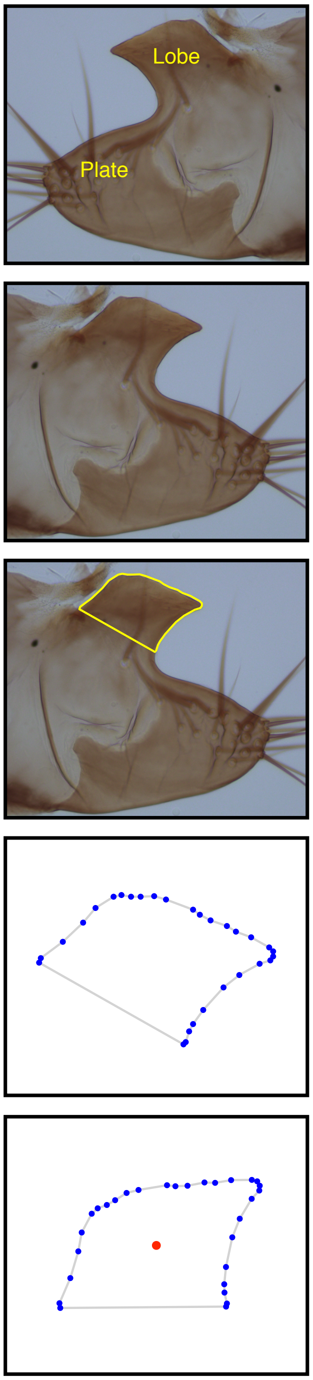


**STEP 1**
Take image of posterior lobe & lateral plate

**STEP 2**
If necessary, flip/rotate image such that "point" of lobe points clockwise

**STEP 3**
Using *ImageJ* define outline of lobe, closing the outline with an artificial baseline

**STEP 4**
Save the series of Cartesian coordinates defining lobe shape/size

**STEP 5**
Rotate the coordinate series so the baseline is horizontal, and translate the coordinates so that the centroid of the outline is at the origin (•)
