# Supplementary material for: Mapping QTL Contributing to Variation in Posterior Lobe Morphology between Strains of Drosophila melanogaster
Source: PLoS One. 2016 Sep 8;11(9):e0162573. doi: 10.1371/journal.pone.0162573 (PMC5015897; doi:10.1371/journal.pone.0162573)

Supplementary Figure S2. Genotypes of recombinant individuals. Each of the recombinant individuals (rows) is given a genotype for a set of non-overlapping 250 kb windows along the genome (columns). Windows where no genotype call was made are shown in white. The top two rows of the image represent the homozygous parental T7 and SS parental strains, which receive homozygous calls (red and blue, respectively) for all windows genotyped. The thick black line at the bottom of the image depicts which genotyped windows were used as markers for QTL mapping.

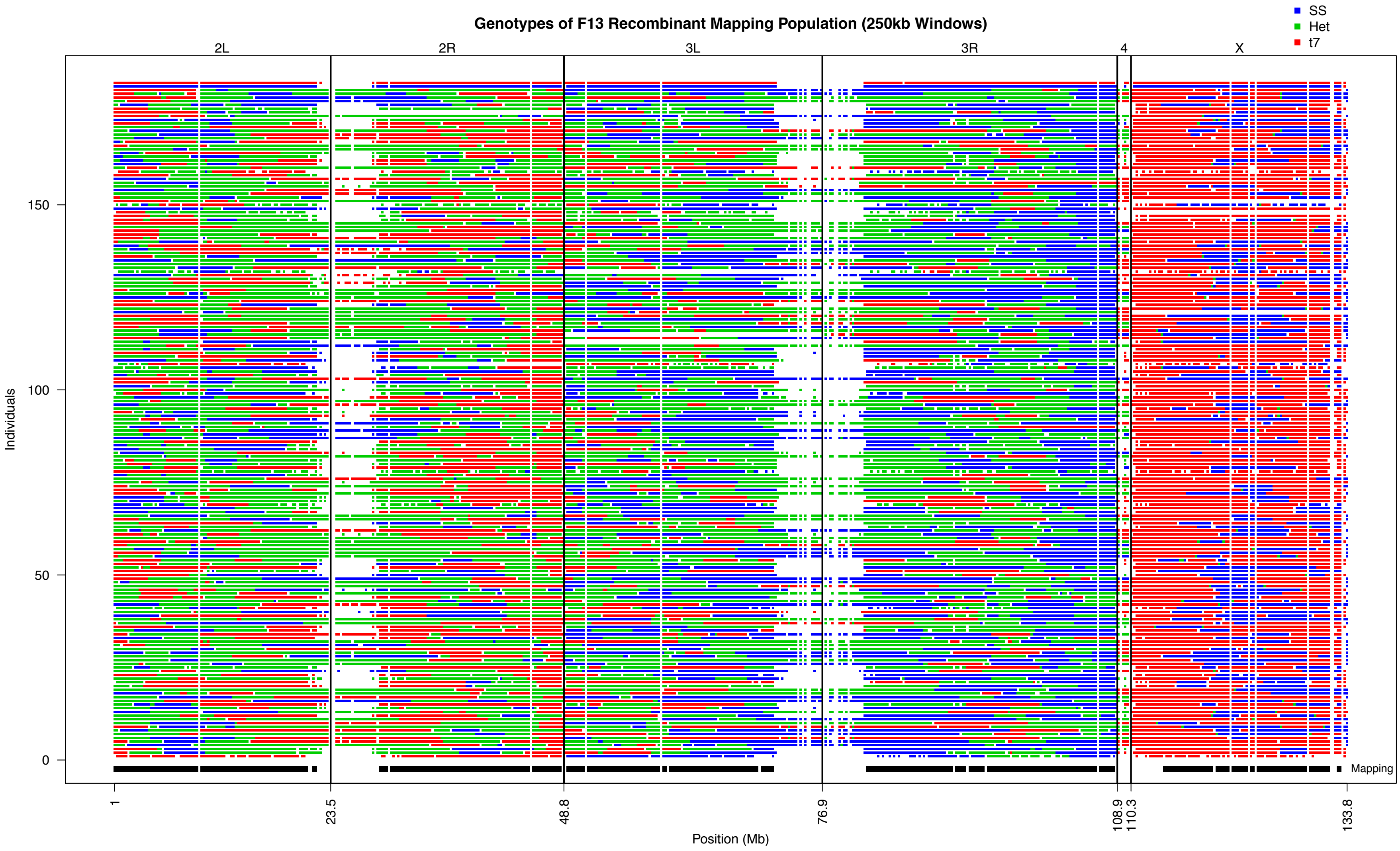

Supplement: S2 Fig — Each of the recombinant individuals (rows) is given a genotype for a set of non-overlapping 250 kb windows along the genome (columns). Windows where no genotype call was made are shown in white. The top two rows of the image represent the homozygous parental T7 and SS parental strains, which receive homozygous calls (red and blue, respectively) for all windows genotyped. The thick black line at the bottom of the image depicts which genotyped windows were used as markers for QTL mapping. (PDF) [file pone.0162573.s003.pdf]
