# Supplementary material for: Mapping QTL Contributing to Variation in Posterior Lobe Morphology between Strains of Drosophila melanogaster
Source: PLoS One. 2016 Sep 8;11(9):e0162573. doi: 10.1371/journal.pone.0162573 (PMC5015897; doi:10.1371/journal.pone.0162573)

Supplementary Figure S3. Clustering recombinants based on their genotype. The number of differences in genotype was calculated between every pair of recombinants for the set of window-based markers on the five major chromosome arms (X, 2L, 2R, 3L, 3R). This dissimilarity, or mismatch matrix was then subjected to hierarchical clustering using the 'hclust' function in R [GenoTree <- hclust(as.dist(MismatchMatrix),method="average")]. The plot below depicts the resulting dendrogram, with recombinants highlighted based on genotyping data collection method (black, MSG; red, WGS). There is no clear clustering of animals genotyped with the same method.

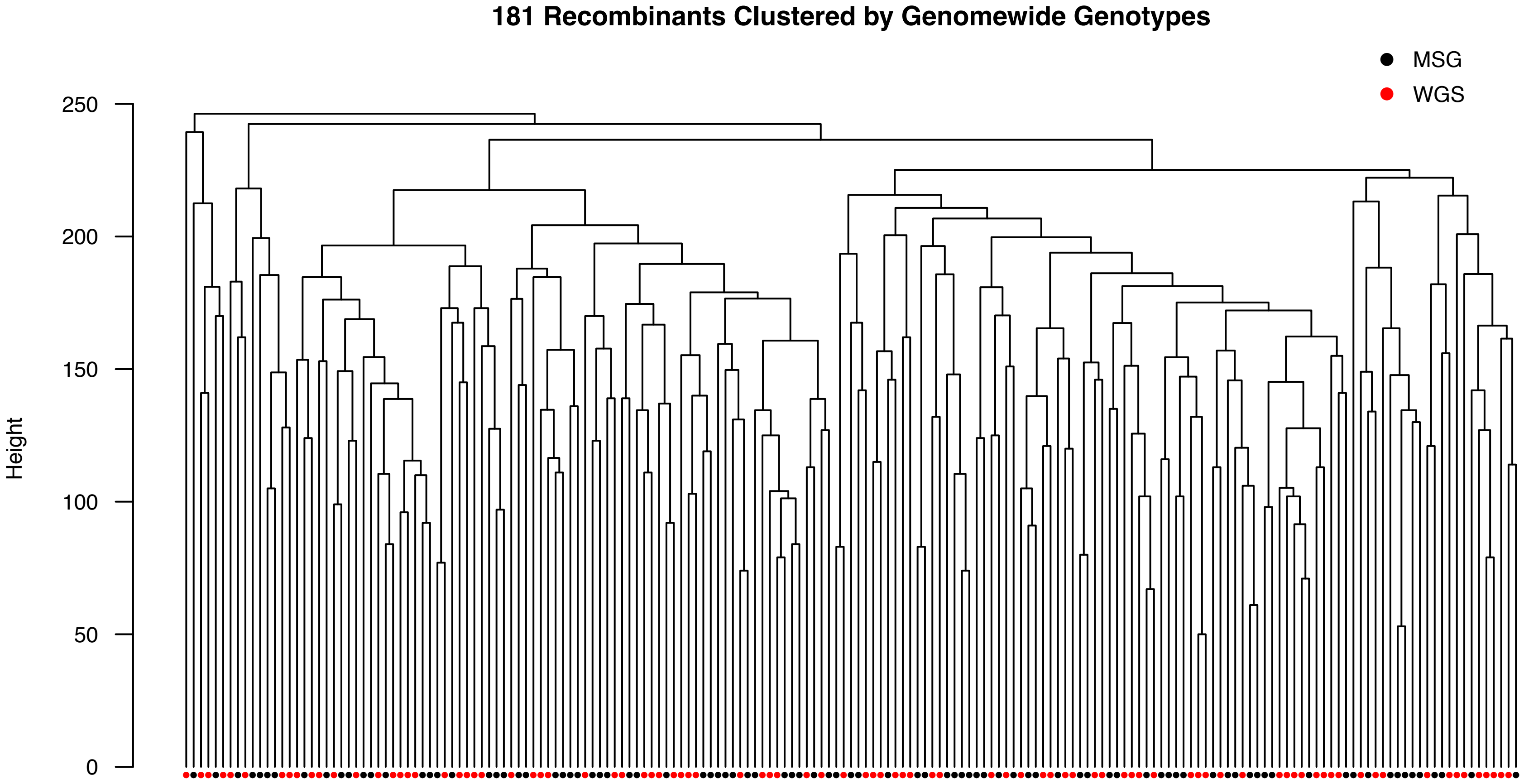

Supplement: S3 Fig — The number of differences in genotype was calculated between every pair of recombinants for the set of window-based markers on the five major chromosome arms (X, 2L, 2R, 3L, 3R). This dissimilarity, or mismatch matrix was then subjected to hierarchical clustering using the 'hclust' function in R (GenoTree <- hclust(as.dist(MismatchMatrix),method = "average")). The plot depicts the resulting dendrogram, with recombinants highlighted based on genotyping data collection method (black, MSG; red, WGS). There is no clear clustering of animals genotyped with the same method. (PDF) [file pone.0162573.s004.pdf]
