# Supplementary material for: Mapping QTL Contributing to Variation in Posterior Lobe Morphology between Strains of Drosophila melanogaster
Source: PLoS One. 2016 Sep 8;11(9):e0162573. doi: 10.1371/journal.pone.0162573 (PMC5015897; doi:10.1371/journal.pone.0162573)

Supplementary Figure S4. Parental allele frequencies in the recombinant mapping population. Each point depicts the frequency of the T7 allele in the panel of 181 recombinant individuals at one of the 250kb markers. Windows with T7 allele frequency above 0.8 or below 0.2 are highlighted in red.

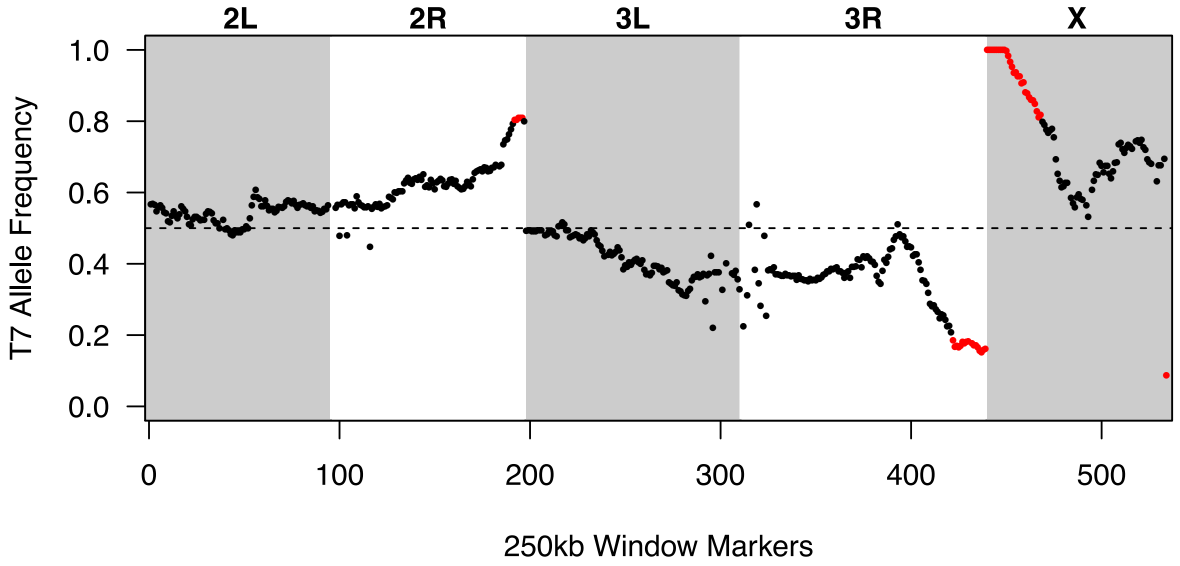

Supplement: S4 Fig — Each point depicts the frequency of the T7 allele in the panel of 181 recombinant individuals at one of the 250kb markers. Windows with T7 allele frequency above 0.8 or below 0.2 are highlighted in red. (PDF) [file pone.0162573.s005.pdf]
