# Supplementary material for: Mapping QTL Contributing to Variation in Posterior Lobe Morphology between Strains of Drosophila melanogaster
Source: PLoS One. 2016 Sep 8;11(9):e0162573. doi: 10.1371/journal.pone.0162573 (PMC5015897; doi:10.1371/journal.pone.0162573)

Supplementary Figure S5. Subset of posterior lobes sorted by the value of PC1 shape phenotype. Four lobes from each parental strain are presented (SS, blue; T7, red), along with 88 lobes from recombinant individuals (black). The number within each image is the PC1 ($\times 10^4$) value assigned to the lobe.

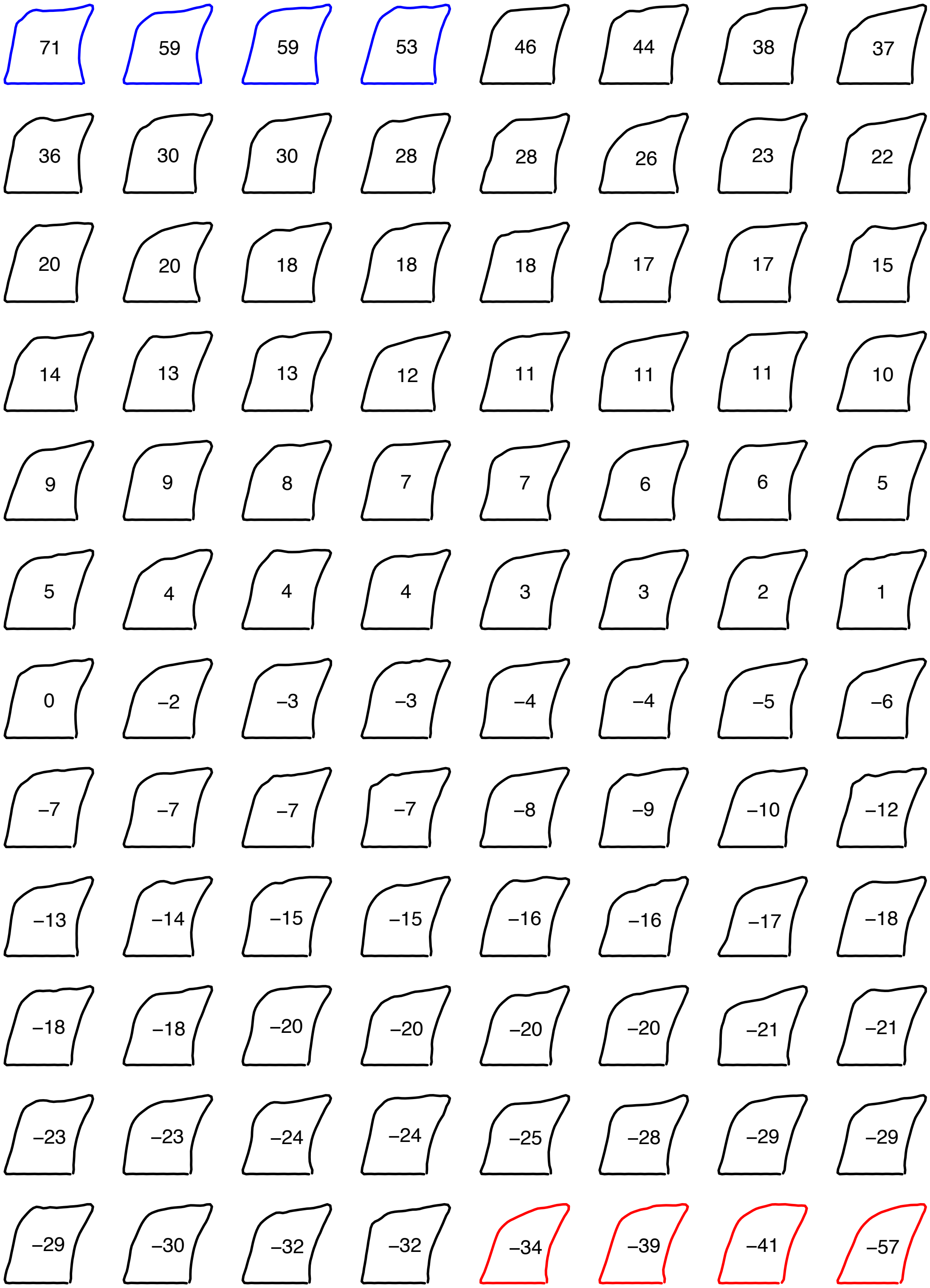

Supplement: S5 Fig — Four lobes from each parental strain are presented (SS, blue; T7, red), along with 88 lobes from recombinant individuals (black). The number within each image is the PC1 (× 104) value assigned to the lobe. (PDF) [file pone.0162573.s006.pdf]
