# Supplementary material for: Mapping QTL Contributing to Variation in Posterior Lobe Morphology between Strains of Drosophila melanogaster
Source: PLoS One. 2016 Sep 8;11(9):e0162573. doi: 10.1371/journal.pone.0162573 (PMC5015897; doi:10.1371/journal.pone.0162573)

Supplementary Figure S6. Effect plots for all six PC1 and PC2 QTL. The phenotype data for the recombinants is plotted against their genotypes at the six QTL peaks. For each genotypic class, the mean (+/– 1-SE) phenotype is also presented.

PC1 QTL:

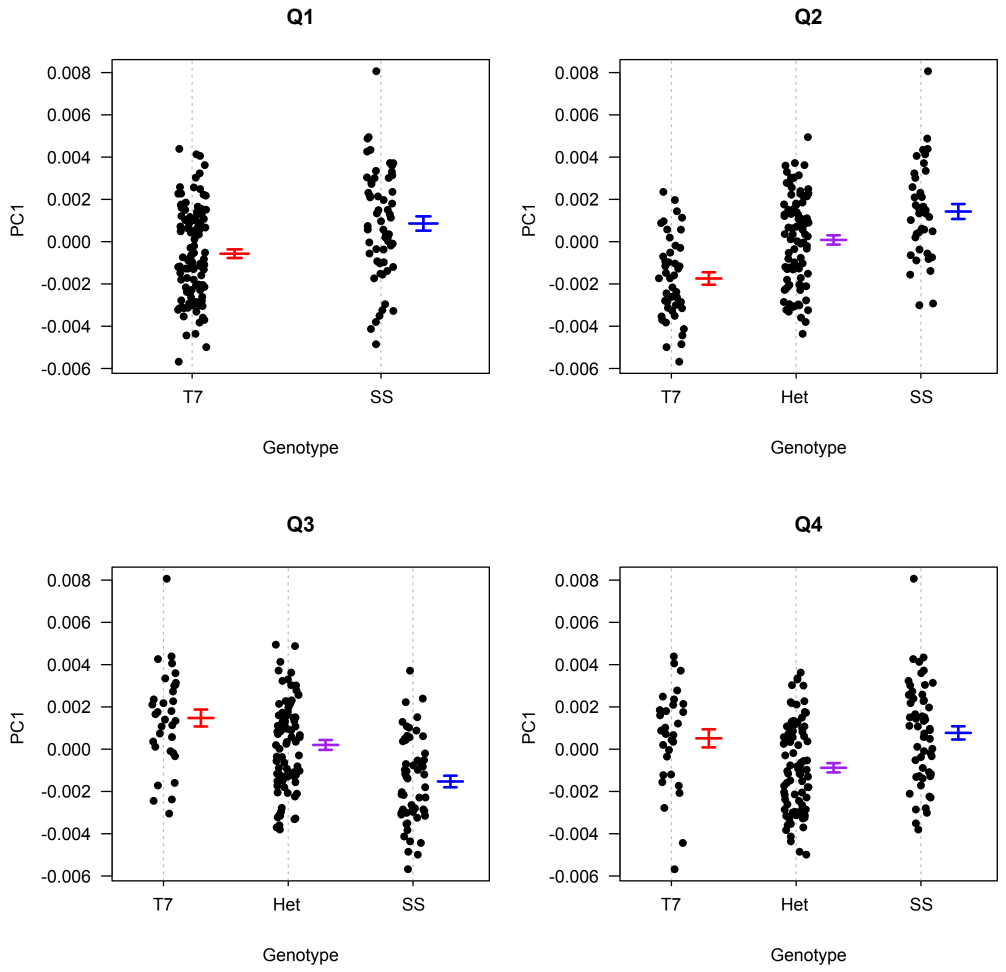

PC2 QTL:

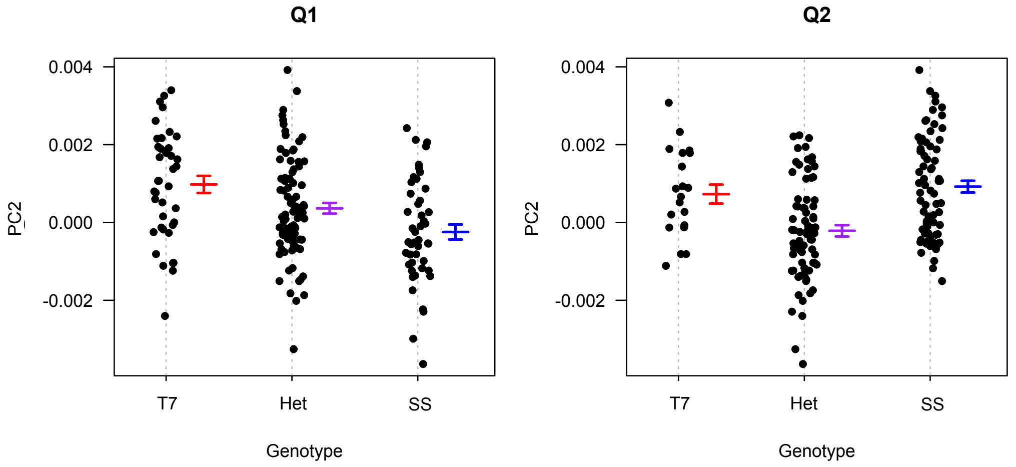

Supplement: S6 Fig — The phenotype data for the recombinants is plotted against their genotypes at the six QTL peaks. For each genotypic class, the mean (+/– 1-SE) phenotype is also presented. (PDF) [file pone.0162573.s007.pdf]
