# Supplementary material for: Mapping QTL Contributing to Variation in Posterior Lobe Morphology between Strains of Drosophila melanogaster
Source: PLoS One. 2016 Sep 8;11(9):e0162573. doi: 10.1371/journal.pone.0162573 (PMC5015897; doi:10.1371/journal.pone.0162573)
Supplement: S5 Table — (PDF) [file pone.0162573.s012.pdf]

Supplementary Table S4. Positions of QTL mapped for all 7 traits.

Trait	QTL [a]	LOD	Chr	Interval (cM) [b]	Interval (Mb) [b]
lobeArea	Q1 [3]	5.2	3L	141-158	17.4-21.7
lobeHeight	Q1 [1]	2.7	X	1-45	3.5-9.3
lobeHeight	Q2 [2]	4.6	2L	25-45	3.3-5.3
lobeHeight	Q3 [3]	4.4	3L	142-166	3L:17.8-3R:8.3 [c]
lobeWidth	Q1 [2]	5.8	2L	12-51	13.8-16.4
lobeWidth	Q2	3.8	2L	135-149	2.0-5.6
HWratio	Q1 [1]	2.7	X	1-90	3.5-12.5
HWratio	Q2 [2]	10.6	2L	36-46	4.0-5.4
PC1	Q1	2.9	X	106-165	13.6-19.4
PC1	Q2 [2]	9.8	2L	36-50	4.1-5.6
PC1	Q3	8.6	3L	85-119	10.4-13.7
PC1	Q4	4.6	3R	262-277	21.7-23.7
PC2	Q1	4.3	3L	34-72	4.5-9.5
PC2	Q2 [3]	6.0	3L	146-155	18.7-21.2
PC3	Q1	4.2	2L	64-85	7.0-9.5

[a] The superscript numbers indicate those QTL intervals that physically overlap. For instance, both QTL labeled "1" overlap with each other. QTL without a superscript label are uniquely found for a single trait.

[b] QTL intervals are defined by 2-LOD drops from the peaks. Genetic distance is given on the expanded map (map distances on the typical *D. melanogaster* genetic map will be approximately 3 times shorter.) Physical distance is provided relative to Release 6 of the genome.

[c] This QTL interval overlaps the chromosome 3 centromere, so the proximal and distal ends of the interval are on different chromosome arms.
